# Regeneration of glycocalyx by heparan sulfate and sphingosine 1-phosphate restores inter-endothelial communication

**DOI:** 10.1371/journal.pone.0186116

**Published:** 2017-10-12

**Authors:** Solomon A. Mensah, Ming J. Cheng, Homa Homayoni, Brian D. Plouffe, Arthur J. Coury, Eno E. Ebong

**Affiliations:** 1 Department of Bioengineering, Northeastern University, Boston, Massachusetts, United States of America; 2 Department of Chemical Engineering, Northeastern University, Boston, Massachusetts, United States of America; 3 Department of Neuroscience, Albert Einstein College of Medicine, New York, New York, United States of America; Rutgers University, UNITED STATES

## Abstract

Vasculoprotective endothelium glycocalyx (GCX) shedding plays a critical role in vascular disease. Previous work demonstrated that GCX degradation disrupts endothelial cell (EC) gap junction connexin (Cx) proteins, likely blocking interendothelial molecular transport that maintains EC and vascular tissue homeostasis to resist disease. Here, we focused on GCX regeneration and tested the hypothesis that vasculoprotective EC function can be stimulated via replacement of GCX when it is shed. We used EC with [i] intact heparan sulfate (HS), the most abundant GCX component; [ii] degraded HS; or [iii] HS that was restored after enzyme degradation, by cellular self-recovery or artificially. Artificial HS restoration was achieved via treatment with exogenous HS, with or without the GCX regenerator and protector sphingosine 1- phosphate (S1P). In these cells we immunocytochemically examined expression of Cx isotype 43 (Cx43) at EC borders and characterized Cx-containing gap junction activity by measuring interendothelial spread of gap junction permeable Lucifer Yellow dye. With intact HS, 60% of EC borders expressed Cx43 and dye spread to 2.88 ± 0.09 neighboring cells. HS degradation decreased Cx43 expression to 30% and reduced dye spread to 1.87± 0.06 cells. Cellular self-recovery of HS restored baseline levels of Cx43 and dye transfer. Artificial HS recovery with exogenous HS partially restored Cx43 expression to 46% and yielded dye spread to only 1.03 ± 0.07 cells. Treatment with both HS and S1P, recovered HS and restored Cx43 to 56% with significant dye transfer to 3.96 ± 0.23 cells. This is the first evidence of GCX regeneration in a manner that effectively restores vasculoprotective EC communication.

## Introduction

The vasculoprotective endothelial cells (ECs) exhibit a number of behaviors that include regulation of vascular permeability and inflammation along with control of vascular tone [1]. An important contributor to these functions is the EC membrane-anchored, mesh-like extracellular matrix; a sugar coated known as the glycocalyx (GCX) [2, 3]. The location and anchoring of the GCX enables EC sensitivity to the extracellular microenvironment conditions [4], which ECs transduce into specific biological behaviors in a temporal and spatial manner [5, 6].

GCX composition is actively regulated by EC through continuous shedding and synthesis [7–12]. At healthy vascular sites, shedding and synthesis are balanced and the intact GCX can transmit signals into the cell to trigger vasculoprotective cell function [4, 13]. At diseased vascular sites, shedding exceeds synthesis leading to increased GCX permeability and/or reduced thickness [14–16]. Consequently, cell signal transmission becomes dysfunctional [4] in a manner that is permissive of disease. Regeneration of shed GCX to reverse this dysfunctional cell signaling and prevent disease was the focus of the study described herein. Specifically, we sought to functionally regenerate the most abundant GCX constituent, heparan sulfate (HS).

Proposed HS regeneration approaches include its replacement, structural stabilization, competitive binding, and synthesis enhancement [17]. Previously, our group and others successfully replaced shed HS with commercial HS [18]or sulodexide [19, 20], a compound containing both heparin and dermatan sulfate. In other studies, a non-animal heparan sulfate-like polysaccharide, called rhamnan sulfate, was used as a vascular EC HS mimetic [17, 21–23]. For non-vascular applications, semi-synthetic and heparin-like pentosane polysulfate was used as an HS substitute [24]. Compared to HS replacement strategies, structural stabilization of HS is less common, and was only recently achieved through employment of sphingosine 1-phosphate (S1P) [25, 26].

We considered the previous HS regeneration studies, and focused our approach by regenerating HS using exogenous HS. We did not combine exogenous HS alone and not in combination with dermatan sulfate as it exists in sulodexide, because dermatan sulfate is not among the GAGs that are naturally present in vascular EC GCX [27]. GCX susceptibility to collapse, shedding, or other damage in the presence of destabilizing chemical and mechanical factors [28] was taken into consideration. To minimize GCX instability, we combined HS with S1P to prevent GCX degradation during the regeneration process [25, 26, 29]. In summary, we examined EC with [i] intact HS; [ii] enzymatically degraded HS; and [iii] HS that was artificially regenerated after enzyme degradation.

In previous studies, described above, efficacy of HS regeneration was tested by examining transendothelial permeability and vascular inflammation as markers of HS-dependent cell function [18–20, 25, 30]. Few other important EC functions were tested [20]. To help advance efforts to therapeutically rebuild the GCX in a functional manner, we assessed the efficacy of HS regeneration by probing gap junctions, which are of great interest due to their complexity and mediation of many vasculoprotective EC functions [31–33]. Gap junctions in EC are formed by connexin (Cx) proteins of various isoforms. Cx43 is the most abundantly expressed connexin in cultured EC [34]making it the focus of many previous studies, as well as the present investigation, via immunocytochemical characterization of Cx43 expression at EC borders. Based on prior work by Thi et al [4], we expect to find that HS regeneration will stabilize Cx expression, supporting open gap junction communication. Thi and colleagues noted that HS-bound syndecan and F-actin are connected, and F-actin is linked to the intercellular junction complex via zona occludin 1 (ZO-1) [35], which plays a role in docking the Cx gap junction proteins at the cell membrane [36–38]. Thi also noted coordinated F-actin stress fiber formation and ZO-1 and Cx43 reorganization in response to flow stimuli, only when HS is intact and not when it is degraded [4]. This prior work [4] suggests that reinforcing HS to neutralize the effect of its shedding from the GCX will maintain Cx43 expression and open gap junctional communication. To confirm this, our study determined HS regeneration efficacy by measuring EC-to-EC spread of gap junction permeable Lucifer Yellow dye as an indicator of the activity level of Cx-containing gap junctions.

Shedding of GCX and its component HS, as well as alterations in gap junctional communication, have all been connected to the onset and the progression of many vascular diseases including atherosclerosis [39–43]. Therefore, the results of this study will enhance our understanding of vascular disease mechanisms and may introduce a new approach to restoring vascular health.

## Materials and methods

### General methods

The general design of this investigation is summarized in Table 1.

**Table 1:** Summary of experimental design.

		GLYCOCALYX CONDITIONS					
		Control	Degraded	Regenerated			
		Untreated	Hep III	Self-Recovery (24 hrs after HepIII)	HS treated (16 hrs after HepIII)	S1P treated (16 hrs after HepIII)	HS/S1P treated (16 hrs after HepIII)
**CULTURE CONDITIONS**	**DMEM + 1% P/S**	x	x	x	x	x	x
	**10% FBS**	x	x	x	x	x	x
**PRE-TREATMENT** **(2 hours)**	**DMEM + 1% P/S**	x	x	x	x	x	x
	**1% BSA**	x	x	x	x	x	x
	**2.5x10**^**-6**^ **IU Hep III**		x	x	x	x	x
**POST-TREATMENT**	**DMEM + 1% P/S**	x	x	x	x	x	x
	**1% BSA**	x	x	x	x	x	x
	**59 mg/ml HS**				x		x
	**10 μM S1P**					x	x
**ASSAYS OF EC FUNCTION**	**HS Expression**	x	x	x	x	x	x
	**Cx43 Expression at EC Borders**	x	x	x	x	x	x
	**Gap Junction Communication (Dye Transfer)**	x	x	x	x	x	x

Abbreviations: Hep III is heparinase III; HS is heparan sulfate; S1P is sphingosine 1-phosphate; DMEM is Dulbecco’s Modified Eagle Medium; P/S is penicillin/streptomycin; FBS is fetal bovine serum; BSA is bovine serum albumin; IU is international units; Cx43 is connexin isoform 43.

### Cell culture

Rat fat pad ECs (RFPEC) [44] at passages 20 to 39 were offspring of cells isolated from rat epididymal fat pad [44] and provided by Dr Mia Thi of Albert Einstein College of Medicine (Bronx, NY). They were used because they exhibit abundant glycocalyx compared to other cell types and also respond to shear stress like other endothelial cells [4, 45]. The RFPEC are immortalized, making it possible for us to use the late passages. RFPEC were seeded on 12–14 mm diameter and 0.13–0.17 mm thick glass coverslips at a seeding density of 15,000–20,000 cells/cm^2^. Cells were cultured in Dulbecco’s Modified Eagle Medium (DMEM, Invitrogen, USA) with 1% penicillin-streptomycin (PS) and 10% fetal bovine serum (FBS, Gibco Life Technologies). Cells were maintained in humidity at 37°C and 5% CO_2_. RFPEC reached full confluence in 3 days.

Cultured RFPEC produce thick and robust GCX in this time period, which is an advantage. At 3 days post-seeding the RFPEC culture medium was supplemented with 1% BSA in place of FBS, to stabilize the GCX during preservation and immunolabeling (described below). For enzyme treatment experiments, 25 micro-international units per milliliter (μIU/ml) of heparinase III (Hep III; IBEX, Canada) were added to BSA-containing culture media for 2 hours to degrade HS from the GCX [46]. HepIII was washed out, followed by 16-hour incubation of RFPEC in regular BSA-containing media. In self-recovery experiments, RFPEC GCX was left to recover for 24 hours after enzyme degradation. The time frame was chosen in accordance with the 20-hour time period that is required for HS restoration to occur on the surface of ECs [47]. For HS recovery experiments, exogenous HS and/or S1P were applied for a critical 16-hour time frame to accommodate our communication test as an assessment of functional glycocalyx recovery. Our group previously showed that, upon external stimulation, induced communication (transfer of gap junction permeable Lucifer yellow dye) between neighboring endothelial cells is time dependent exhibiting a substantial increase in communication in 16 hours compared to a low level of communication in times limited to 5 hours [6]. After HS was degraded with HepIII it was replaced by a 16-hour feeding of RFPEC with 59 μg/ml exogenous porcine mucosal HS (Celsus, Cincinnati, OH), based on published serum concentration of HS [43]. In Sphingosine 1-phosphate experiments, culture media containing enzyme was substituted for media containing either 10μM of S1P (Sigma-Aldrich) or a combination of 59 μg/ml of HS and 10μM of S1P for 16 hours after enzyme degradation.

Below we describe how GCX structure, connexin expression, and gap junctional coupling were characterized in RFPEC with intact GCX, HS degraded GCX, or different modes of GCX repair.

### Scanning electron microscopy

RFPECs, still adherent on glass, were fixed for 1 hour in a mixture of 2% paraformaldehyde, 2% glutaraldehyde, and 0.1 M cacodylate buffer, with or without 0.15% ruthenium red, at a pH of 7.4. After fixation, RFPEC were washed three times at 5 to 10 minute intervals, prior to being incubated for 1 hour in 0.15 M cacodylate buffer containing 1% osmium tetroxide. Following another cacodylate buffer wash cycle, RFPEC were dehydrated using a graded series of ethanol concentrations that included 30%, 50%, 70%, 85%, and three times at 100%, each for 5 minutes at a time. The dehydration series was followed by critical point drying from CO2. The RFPEC on glass were then attached to sample mounts using double-sided carbon adhesive and coated with 5 nm platinum using a Cressington 208 HR sputter coater. Imaging was performed using a Hitachi S-4800 scanning electron microscope at low accelerating voltage of 3 kV and a magnification of 3000x.

### Immunostaining

RFPEC have high affinity to most antibodies that are available for performing immunocytochemistry [46]. For heparan sulfate (HS), RFPEC monolayers treated with 2% paraformaldehyde/1% glutaraldehyde in phosphate buffered saline (PBS) for fixation and treated with 2% goat serum in PBS to block non-specific ligands, were stained for three nights at 4°C with a 1:100 10E4-epitope HS antibody (Amsbio).

As specified by manufacturer, the HS antibody reacts with many types of heparan sulfate. The reactivity of the HS antibody is abolished after treatment with Hep III, indicating antibody specificity [48, 49]. For secondary detection of HS, Alexa Flour 488 conjugated goat anti-mouse IgG/IgM (H + L; Life Technologies) secondary antibody was used. RFPEC to be processed for connexin 43 (Cx43) were fixed in 4% paraformaldehyde, permeabilized in 0.2% Triton X-100 (Fisher), and blocked in 5% goat serum combined with 0.2% Triton X-100. Connexin 43 staining was performed overnight at 4°C with 1:100 mouse monoclonal Cx43 antibody (Millipore). The manufacturer indicates that anti-Cx43 corresponds to amino acids 131–142 of human Cx43 and is homologous with rat Cx43 (Millipore). Secondary Cx43 detection was done with Alexa Flour 488 conjugated goat anti-mouse IgG. Control RFPEC samples in which anti-HS or anti-Cx antibodies were omitted prior to the application of secondary antibodies did not exhibit immunofluorescence. These controls confirmed that the staining observed in the RFPEC is not artefactual.

### Confocal immunofluorescence microscopy imaging and analysis

Alexa Fluor 488 fluorescent RFPEC were mounted with Vectashield containing DAPI (Vector Labs) and imaged with a Zeiss LSM 700 laser scanning confocal microscope. HS and Cx43 were imaged at 63X magnification (oil objective) and 40X magnification (oil objective), respectively. Lasers with excitation wavelengths of 490 nm (for HS or Cx43) and 350 nm (for DAPI) were used to obtain XY-plane slices. Laser gain and transmission were kept below fluorophore saturation levels. Confocal slice intervals were 0.2 μm for HS and 0.7 μm for Cx43. Slices were Z-projected using NIH ImageJ software.

For further HS analysis, from the en face view of the 490 nm channel Z–projection, ImageJ measured and divided the area of fluorescence by the area of the total field of view to obtain a percent value. This percentage represented the amount of RFPEC that was covered by GCX. Cross-sectional images were qualitatively assessed to ensure HS presence on the RFPEC surface and not inside the cells.

To complete our analysis of Cx43, images of multiple fields were collected to form tiles capturing a 10,0000 μm^2^ field of view. From the en face view of the 490 nm channel Z–projection, ImageJ randomly selected nine cells (S2A Fig). The ImageJ freehand tool was used to outline the border of each of the nine cells, and to measure each cell perimeter. The length of the perimeter portion that showed Cx43 fluorescence was also measured. The Cx43 length was divided by the perimeter length to determine the percentage of Cx43 distribution along the perimeter of the cell.

### Dye transfer assay, fluorescence microscopy, & image analysis

Gap junction functionality was assessed by loading Lucifer Yellow in RFPEC, using a scratch technique [50], and observing the extent of Lucifer Yellow transfer from loaded cells to neighboring cells (S2B Fig). Lucifer Yellow has a molecular weight of 457.3 and can only enter cells via broken membranes or via gap junctions. The scratch loading technique involved pre-incubation of RFPEC (for 1 min) with calcium and magnesium free Hanks’ Balanced Salt Solution (HBSS; Life Technology) containing 5 mg/ml Lucifer Yellow dye (Life Technology). We then used a 5μm diameter tip scribe (Ted Pella, Inc, USA) to carefully create a straight scratch in the RFPEC monolayer and to allow the dye to enter the scratched cells. After the dye was loaded, it spread via open gap junctions to adjacent intact cells, with the extent of Lucifer Yellow spread reaching a plateau by 10 minutes after loading. At that point, excess dye was washed out and RFPEC were imaged at 10X magnification (dry objective) using a Zeiss Observer Z1 fluorescence microscope. From recorded images of dye transfer after scratch loading, ImageJ randomly selected dye spread locations that would be quantified along the scratch. At those random locations, lines perpendicular to the scratch axis were drawn along the perpendicular lines, the intact fluorescent cells (scratched cells excluded) were counted. The resultant values were taken to represent the extent of Lucifer Yellow spread across the cell monolayer.

### Statistics

Data were obtained from 3–5 separate experiments. Per condition, duplicate samples were examined. For HS samples we obtained 5 data points, for Cx43 9 data points, and for Lucifer Yellow 20 data points. Data was reduced to mean ± SEM. GraphPad Prism software was used to analyze the data via one-way ANOVA and Bartlett’s statistical correction test, which is sensitive to deviation from normality. Differences in means were statistically significant at p < 0.05. Statistical significance of differences in means is specified in the Figures and captions.

## Results

### HS expression in intact GCX, enzyme degraded GCX, and repaired GCX conditions

We examined the membrane-attached extracellular matrix of RFPEC cultured at passages 20 to 39 on 0.13–0.17 mm thick and 12–14 mm diameter round glass. Expression of the GCX and its HS component on the surface of these RFPEC was confirmed using scanning electron microscopy [51] and immunofluorescence confocal microscopy [28, 46], as utilized in previous studies.

**Fig 1:**
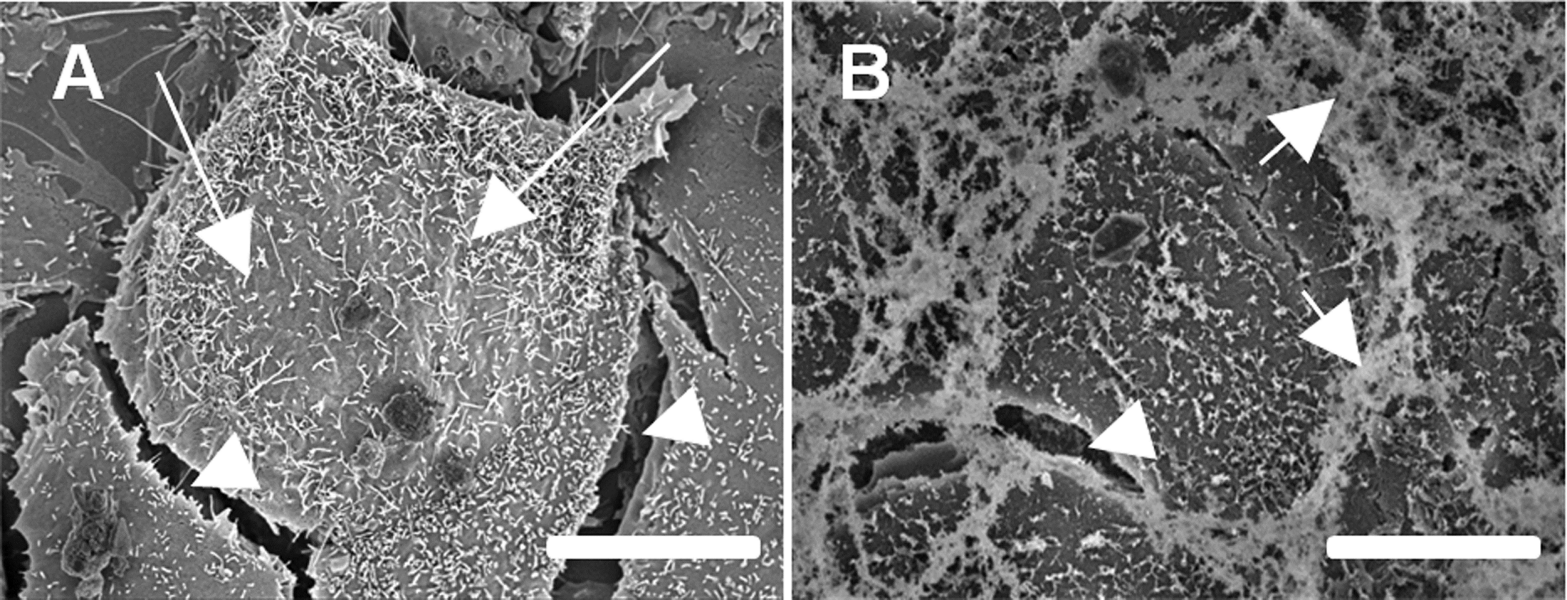
Scanning electron micrographs show brush-like protrusion from the cell surface. **A**. No GCX-specific contrast agent was used. Long arrows point to long, thin extracellular fibrillar structures extending from the surface of the RFPEC plasma membrane. Arrowheads point to intercellular gaps. **B**. Ruthenium red was used as contrasting agent. Ruthenium red specifically binds GAGs and revealed the strong presence of GAGs at the apical surface and at borders of the cell body. Medium length arrows point to ruthenium red at cell borders. Arrowhead points to intercellular gap. Bar = 10 μm.

At baseline conditions (untreated control), where RFPEC were fully confluent and had not yet undergone any treatment, scanning electron micrographs distinctly showed long, thin, extracellular microvilli structures extending from the surface of the RFPEC plasma membrane (Arrows shown in Fig 1A). Ruthenium red staining revealed a large portion of other extracellular structures consisting of GAGs, localized on the apical surface as well as at the junction between cells (Arrow shown in Fig 1B). The GCX collapses during the dehydration process that is required for scanning electron microscopy, complicating quantification of GCX structure [28]. Our scanning electron microscopy experiments revealed a number of intercellular gaps (Arrowheads shown in Fig 1), which we suspected were due to dehydration and which reinforced that this approach would be useful only for GCX localization and structure but not for precise quantification. A correlative microscopy approach that requires less detrimental cell preparation, such as immunofluorescence confocal microscopy, would be required [28].

**Fig 2:**
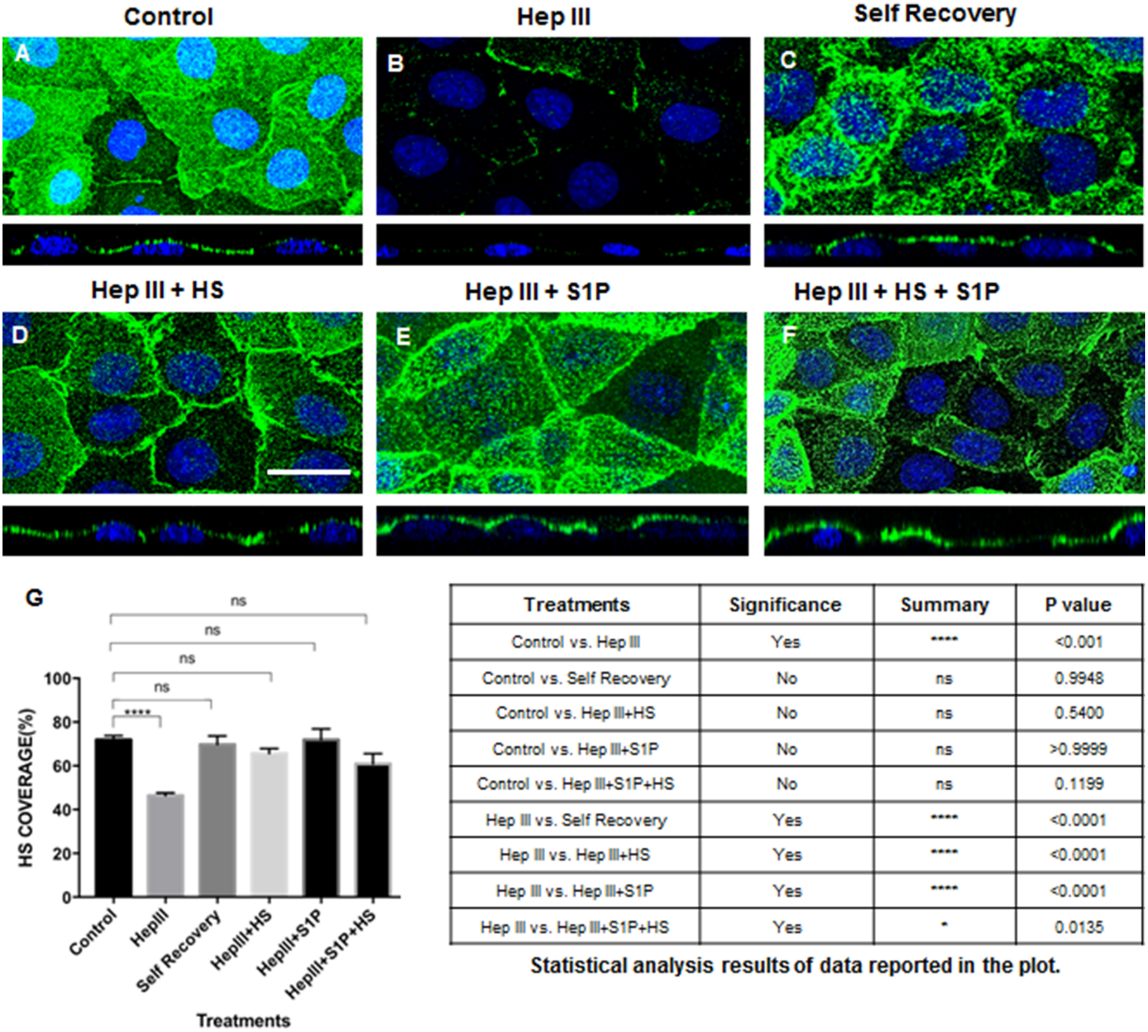
HS expression in intact GCX, enzyme degraded GCX, and repaired GCX conditions. **A**. Untreated (control) cells show intact HS at baseline conditions (green is HS with blue DAPI staining the cell nucleus). **B**. With 25 μIU/ml of Hep III, HS is degraded. **C**. Degraded HS samples were left for 24hrs to allow cells to regenerate HS. **D**. With the addition of HS at a 59 μg/ml concentration, there is a significant recovery of HS back to baseline conditions. Bar = 20 μm, and applies to A through F. **E**. Adding 10μM S1P to enzyme treated samples significantly recovered the expression of HS in GCX, back to baseline conditions. **F**. Combined treatment of exogenous HS and S1P effected HS expression HS by restoring baseline conditions. **G**. Quantification of HS in control, Hep III-treated, self-recovery, and artificial recovery conditions. ANOVA and Tukey post hoc test showed statistical significance as noted in the plot and summarized in the table. The results of various treatment conditions were compared to either control or enzyme (Hep III) conditions.

Confocal micrographs illustrated that one of the GAGs on the apical EC surface included HS, as expected (Fig 2). For baseline conditions in which RFPEC GCX was left intact and untreated, 71.85 ± 1.90% of cell surfaces was covered with HS as indicated in immunofluorescence labelling (Fig 2A and 2G). Control RFPEC samples in which HS antibody were omitted prior to the application of secondary antibodies did not exhibit immunofluorescence (S1A Fig). These controls confirmed that the staining observed in the RFPEC is not artefactual. The effect of enzymatic removal of HS from RFPEC GCX using Hep III is shown in Fig 2B and quantified in Fig 2G. RFPEC treated with a 25 μIU/ml concentration of Hep III for 2 hours, and probed at 16 hours post-enzyme treatment, showed HS coverage of 46.4 ± 1.20% which is statistically significantly less than in untreated control samples (Fig 2G). As a positive control, after enzyme degradation the RFPEC were allowed to recover for 24 hours and self-regenerate their HS coverage. The 24-hour time period for self-regeneration was selected because it is established that HS restoration on the surface of ECs requires 20 hours in static conditions [47]. This cellular self-regeneration resulted in restoration of HS coverage to 69.79± 3.92% (Fig 2C and 2G), which was statistically similar to baseline conditions and statistically greater than enzyme treatment conditions (Fig 2C and 2G). We found that restoration of HS by artificial regeneration could be achieved in a shorter time period (16 hours) than what was required for restoration of HS by cellular self-regeneration. For artificial regeneration of HS, to counteract the effect of Hep III, immediately after Hep III treatment we exposed RFPEC to 59 μg/ml of exogenous HS during the 16-hour period following enzyme treatment. This caused an increase in the coverage of HS to 65.58 ± 2.26% (Fig 2D and 2G). The added HS was also found to be non-toxic for RFPEC (data not shown) and showed up on the cell surface and was not internalized by the cells (Fig 2D orthogonal view). Treatment for 16-hr with 10μM of S1P to neutralize Hep III yielded HS coverage of 71.97 ± 5.02% (Fig 2E and 2G). Delivering 10μM S1P in combination with 59 μg/ml exogenous HS for 16 hours resulted in HS coverage of 60.97 ± 4.59%. Statistical analysis confirmed that artificially recovered HS, via the addition of exogenous HS and/or S1P, was significantly more than HS in enzyme treated conditions and similar to HS in baseline conditions (Fig 2F and 2G).

### Cx43 in intact GCX, enzyme degraded GCX, and repaired GCX conditions

**Fig 3:**
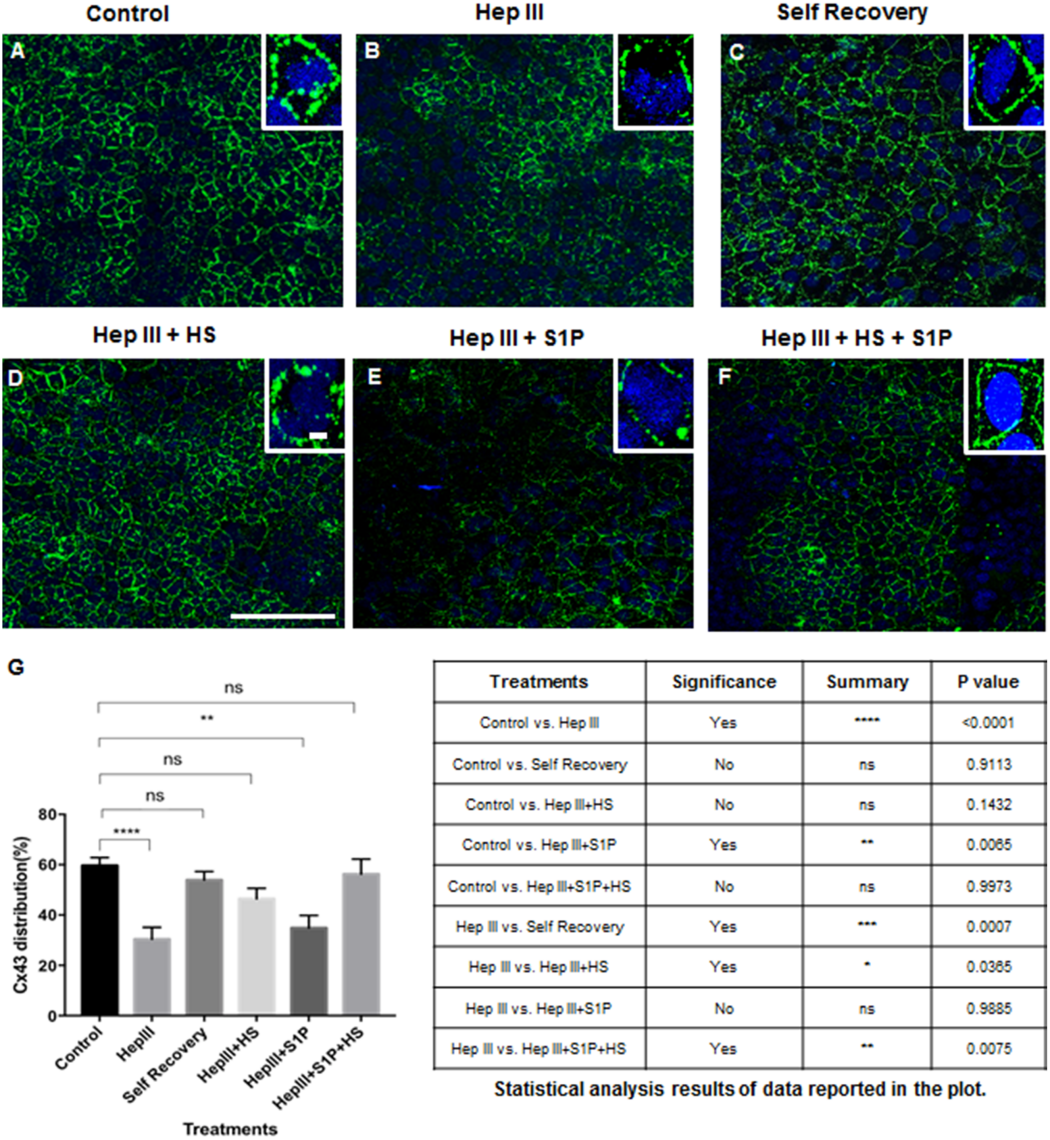
Images and quantification of Cx43 after various modes of GCX recovery. **A—F**. Representative images of Cx43 expression at the monolayer level, with insets clarifying Cx43 distribution at cell borders. **A**. Untreated sample. **B**. After Hep III treatment Cx43 distribution was reduced. **C**. After enzyme treatment, samples were left to self-recover their Cx43 over 24 hours. **D**. Cx43 distribution was partially restored after addition of 59 μg/ml exogenous HS to counteract enzyme treatment. Bar = 100 μm in low magnification image and bar = 2.5 μm in high magnification image, and both apply to A through F. **E**. Addition of 10μM S1P alone did not show a significant restoration of Cx43 expression in comparison of enzyme treated samples. **F**. Combined treatment of HS and S1P after Hep III treatment resulted in expression of Cx43 that matched controls and was greater than in enzyme treated samples. **G**. Quantification of Cx43 distribution at cell borders of RFPEC for control, Hep III-treated, self-recovery, and artificial recovery conditions. ANOVA and Tukey post hoc test showed statistical significance as noted in the plot and summarized in the table. The results of various treatment conditions were compared to either control or enzyme (Hep III) conditions.

To study the effect of GCX HS conditions on Cx-containing gap junctions, we first analyzed the distribution of Cx43 at cell borders in samples of EC with intact GCX HS, degraded GCX HS, and GCX HS recovered by various strategies. Our confocal microscopy studies confirmed that Cx43 is abundantly expressed by RFPEC and localized primarily at the cell borders where functional gap junctional communication takes place. The initial Cx43 distribution in untreated control RFPEC samples was 59.61 ± 3.20% along the perimeter of the cells (Fig 3A and 3G). Specificity of Cx43 immunolabeling was confirmed by omission of Cx43 antibody and incubation of control RFPEC samples with secondary antibody only, which did not result in any detectable immunofluorescence (S1B Fig). Enzymatic removal of HS by using 25 μIU/ml of Hep III resulted in a statistically significant decrease (p<0.05) in the percentage of Cx43 distribution, to 30.40 ± 4.65% (Fig 3B and 3G) which is approximately 30% less than in untreated control samples (Fig 3A and 3G). In RFPEC that were allowed to self-regenerate their HS coverage after enzymatic degradation, Cx43 appeared along 53.89 ± 3.44% of cell borders, which was statistically equivalent to control conditions and statistically greater than enzyme treatment conditions (Fig 3C and 3G). Addition of 59 μg/ml of HS to counteract enzymatic degradation resulted in Cx43 increase to 46.45 ± 4.21%, representing a statistically significant recovery compared to enzyme treated samples and statistical similarity to control conditions (Fig 3D and 3G). Samples with S1P treatment after enzyme degradation presented a Cx43 distribution of 34.81 ± 5.16%, which, to our surprise was not statistically different than enzyme treated samples and statistically less than baseline levels at control conditions (Fig 3E and 3G). Addition of both HS and S1P resulted in an increase in Cx43 distribution to 56.21 ± 6.32%, a statistically significant recovery from enzyme treatment conditions and statistically close to baseline levels (Fig 3F and 3G). This HS recovery due to combined HS and S1P can be attributed primarily to exogenous HS, since S1P alone could not induce HS recovery.

### Gap junction communication is lost with GCX degradation and recovery depends on mode of GCX repair

Having confirmed Cx expression levels per GCX condition, we measured the extent of functional communication via Cx-expressing gap junctions. The opening and closing of the gap junctions was assessed by end-point quantification of the extent of movement of gap junction permeable scratch-loaded Lucifer Yellow dye between neighboring cells, in monolayers of RFPEC with intact GCX and in comparison to monolayers of RFPEC with degraded GCX or after different modes of GCX recovery. In cultured RFPEC monolayers, dye spread (intercellular communication) from the scratch to relatively few neighboring cells was taken to suggest weak communication. Spreading of dye among a relatively large number of cells indicated stronger communication. Dye spread was never observed to extend throughout the entire cell monolayer despite the fact that most RFPEC were expressing Cx43.

**Fig 4:**
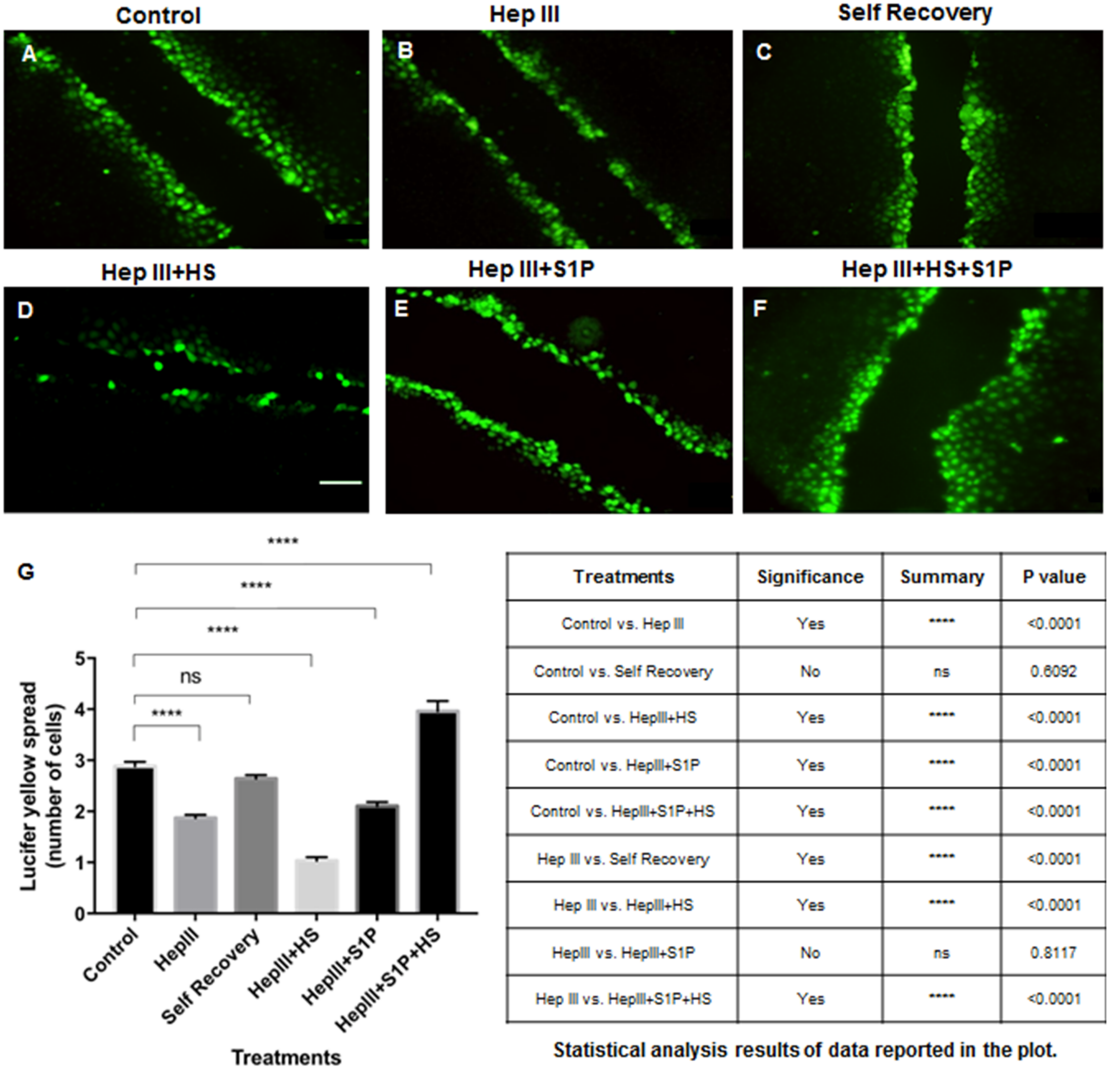
Quantification of Lucifer Yellow dye transfer after various modes of GCX recovery. **A**. Control **B**. With 25 μIU/ml Hep III treatment, the movement of Lucifer Yellow dye across cell junctions was impaired. **C**. Self-recovery of HS, after enzymatic degradation, allowed dye transfer across cell junctions at near baseline levels **D**. Not much Lucifer Yellow dye transfer improvement was observed after recovery of lost HS by addition of 59 μg/ml HS. Bar = 100 μm and applies to A through F. **E**. Addition of 10μM S1P also did not improve cell-to-cell communication. **F**. A combined treatment of RFPEC with both HS and S1P resulted in increase in Lucifer Yellow dye transfer; reversing the effects of enzyme treatment. **G**. Quantification of Lucifer Yellow flux across connexin-containing gap junctions in RFPEC in control, Hep III-treated, self-recovery, and artificial recovery conditions. ANOVA and TUKEY post hoc test showed statistical significance as noted in the plot and summarized in the table. The results of various treatment conditions were compared to either control or enzyme (Hep III) conditions.

As indicated in Fig 4A and 4G, for every cell along the scratch, uploaded Lucifer Yellow dye transferred to 2.88 ± 0.09 neighboring RFPEC in untreated control conditions. Following Hep III removal of HS from RFPEC, we observed a statistically significant decrease in Lucifer Yellow spread, with 1.87 ± 0.06 cells receiving the dye from an adjacent scratch-loaded cell (Fig 4B and 4G). Self-regeneration of HS after enzyme degradation yielded Lucifer Yellow spread of 2.64 ± 0.07 cells which was statistically similar to control conditions and statistically significant recovery from enzyme conditions, as expected (Fig 4C and 4G). In RFPEC that had been exposed to HS degradation enzyme, artificial replacement of HS with the exogenous GAG did not yield any recovery of Lucifer Yellow dye-coupling. We were surprised to discover that, under conditions of HS recovery with exogenous HS, scratch-loaded dye was only transferred to 1.03 ± 0.07 cells (Fig 4D and 4G). This was statistically less than dye transfer in both baseline and Hep III conditions (Fig 4D and 4G). Artificial recovery with only S1P added after enzyme degradation showed a Lucifer Yellow dye spread of 2.06 ± 0.08 cells (Fig 4E and 4G), statistically similar to the dye transfer level under Hep III treatment conditions. We were pleased to find that, after the treatment of RFPEC with combined HS and S1P, 3.96 ± 0.23 cells received Lucifer Yellow dye. This cell number is statistically significantly high when compared to cell number in both untreated control and Hep III-treated conditions (Fig 4F and 4G). This communication data, taken together with the Cx43 data, demonstrates that treatment of ECs with exogenous HS combined with S1P is the best artificial HS regeneration approach for simultaneous recovery of both Cx43 protein and gap junction function.

## Discussion

**Fig 5:**
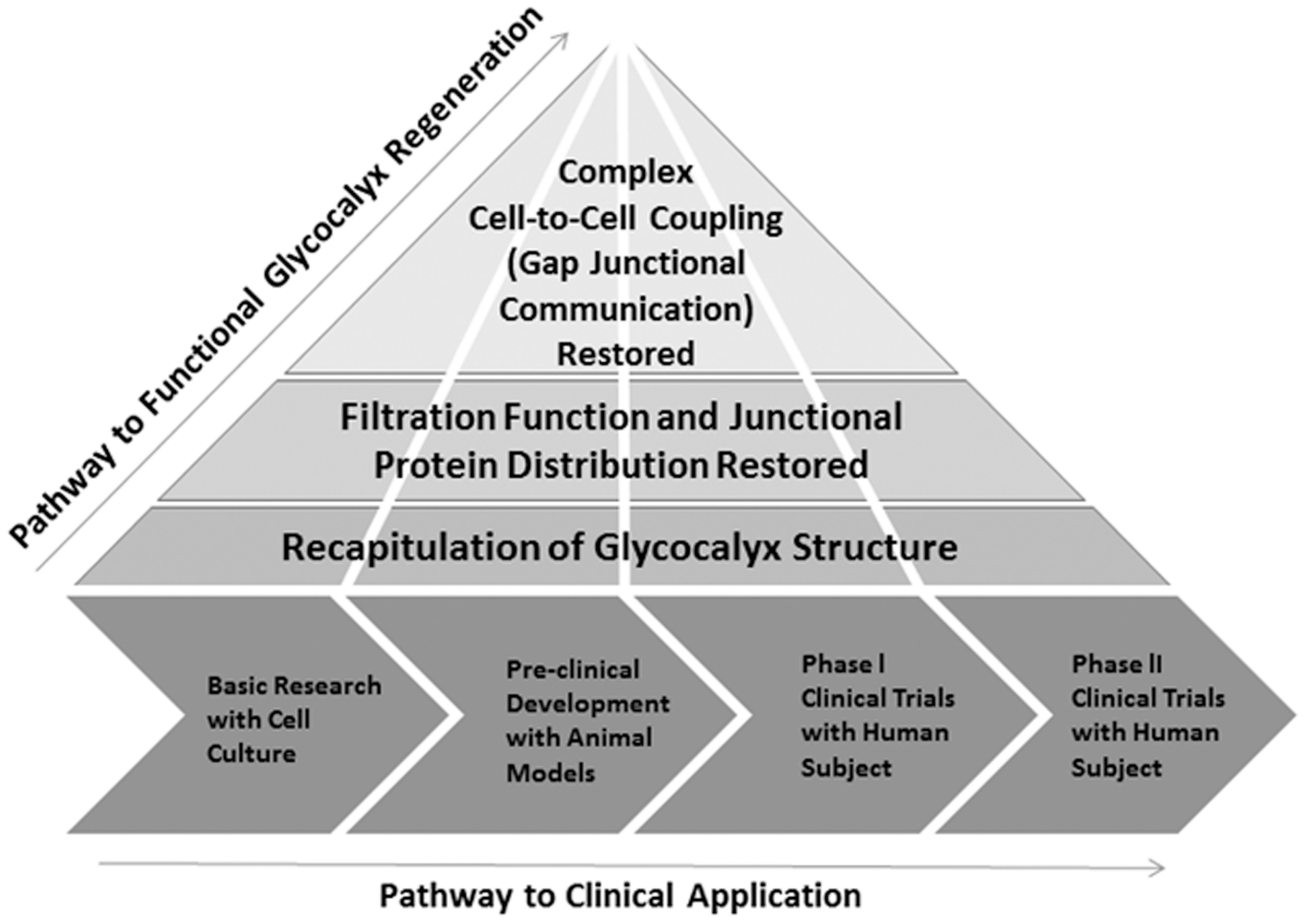
Proposed pathway for eventual translation of functional glycocalyx regeneration to the clinic. According to this pathway, the work presented in this report represents the first step toward clinical translation. This step involves basic cell culture experiments for initial determination of the efficacy of our proposed glycocalyx replacement and stabilization strategy. We used rat endothelial cells to match rat animal experiments that are common for atherosclerosis pre-clinical studies, because the cell culture work will be followed by a series of rat and other animal experiments to test in-vivo performance of the HS/S1P treatment. These cell culture and animal studies are prerequisites for phase I and phase II clinical trials on human volunteers.

We commenced our study by creating cell culture models of degraded and regenerated GCX, using RFPEC as an ideal experimental cell culture system because of the inherent RFPEC expression of abundant GCX that contains substantial HS (Figs 1 and 2). We created a model of degraded GCX via Hep III-induced HS degradation (Fig 2B and 2G), an approach that is consistent with previous work [40, 46, 47]. This enzymatic degradation model mimics diseased conditions for which release of GAGs from the endothelium surface and into the bloodstream have been reported [43, 52, 53]. Our GCX regeneration model, on the other hand, is a new contribution to the GCX research field. To our knowledge, this is the first demonstration that endothelial GCX can be considerably restored in vitro by addition of exogenous HS (Fig 2D). We took advantage of the cell surface GCX capacity to recognize and bind to extracellular GAGs. The concentrations of supplemental HS, 59 μg/ml, was chosen to align with values for the amount of HS found in the arterial blood of patients suffering from global ischemia, reported by Rehm et al. [43]. We anticipated a potential pitfall, in that RFPEC may not incorporate the exogenous HS in a functional manner. Our alternative GCX regeneration strategy was to use S1P to both regenerate and stabilize the GCX [25, 26] after enzymatic degradation and regeneration. It was encouraging to find that feeding RFPEC with the prescribed concentration of exogenous HS alone, S1P alone, or HS together with S1P led to renewed HS expression in the RFPEC surface GCX, at a status that matched control conditions. Although using the rat ECs was ideal because of the abundant GCX that could be easily manipulated, this study is limited by the possibility that the results could be different if other cells were used. For example, using ECs from different vascular beds with potentially different GCX expression patterns could affect the outcomes of this study. Also, primary cell cultures could give different results. Reports of the patterns of HS expression in bovine- and human-derived primary ECs, for example, differ from the RFPEC HS pattern and are regulated by external mechanical stimulation [54]. We are conducting other investigations with primary ECs originating from human vessels and a variety of vascular beds. The other investigations are outside the scope of the study described in this report, and will be published in a separate research article. With this being said, RFPEC are ideal, because they enable translation of this research to preclinical *in vivo* studies in the future (Fig 5). Rat animal models have commonly been used for preclinical translational research to test the ability of new drug treatments to mitigate pro-atherosclerotic factors and attenuate atherogenesis *in vivo* [55]. The RFPEC cultures used in this study are meant to be a species match for the rat animal pre-clinical trials that will be conducted in the future.

GCX importance in key physiological events is well documented in numerous reports on studies of how characteristic cell functions are gained or lost as a consequence of GCX subcomponent degradation [46, 56, 57]. To date, our colleagues Thi et al have been the only group to study GCX regulation of intercellular gap junctional communication, in a very elegant study [4]. They removed the principal GCX component, HS, and demonstrated gap junction protein desensitization to externally applied flow stimuli that would normally displace the protein. In our study, by successfully degrading and subsequently regenerating HS, we created a tool with which to substantially enhance the body of knowledge about GCX role in inter-endothelial gap junction functionality. Similar to previous findings [4], enzymatic degradation of HS reduced Cx43 at the RFPEC perimeter where cell-to-cell communication activity takes placed, by ~50% compared to baseline conditions. Our study is the first to observe significant Cx43 recovery at cell borders, to ~80% of baseline conditions, as a result of simple replacement of HS to counteract enzymatic HS degradation. Combinatorial treatment of RFPEC with HS and S1P resulted in further enhanced Cx43 recovery at cell borders, to ~95% of baseline conditions. These results emphasize the importance of the GCX as a regulator of cell membrane expression of gap junction protein. The expectation is that similar findings can be obtained for active cell communication, which directly depends on Cx expression and mediates other key EC functions that are prominent in vascular health and disease [32, 58].

In contrast to our Cx43 results, changes in active communication (assessed by Lucifer Yellow dye transfer) did not parallel the experimentally induced changes in HS and the GCX. As we expected, removal of HS from RFPEC reduced the spread of dye from scratch-loaded to neighboring cells by ~50%, compared to untreated RFPEC. This reduction was attributed to HS-induced decrease in membrane Cx43 and an associated decline in the number of open gap junction channels. This result further confirmed the fundamental relationship between the GCX and gap junction function. However, with HS regeneration via exogenous HS, gap junction communication remained closed. It is unclear why exogenous HS restoration did not induce gap junction channels to re-open along with renewal of Cx43 expression at cell borders. It is plausible that GCX restoration by exogenous HS does not translate to full repair of the plasma membrane, which is critical for gap junction proteins to be expressed at the membrane [59] at a level that is sufficient for gap junction formation. The most probable explanation is that the experimentally restored GCX may have a configuration of low stability that exerts reduced tension [60] on the trans-membrane core proteins and, consequently, reduced tension on the cytoskeleton. Downstream, we speculate, this low tension may destabilize the cytoskeleton link with the intercellular junction complex. This weak link could result in improper Cx43 alignment and keep connexins from combining to form connexons, block connexon docking to form gap junctions, or prevent gap junction gates from opening. This hypothesis is supported by the results of preliminary studies that actin disruption by Cytochalasin D bars cell-to-cell movement of Lucifer Yellow dye and other ions and molecules (S3 and S4 Figs).

The bioactive agent S1P has been reported to preserve GCX [25, 29, 61], and was recently shown to induce recovery of the HS GCX component in the absence of HS at baseline conditions [29]. The detailed mechanism underlying S1P’s role in GCX preservation and growth is still under investigation. In addition, S1P has been shown to enhance the strength of the intercellular junction complex, in a study that showed S1P causes redistribution of ZO-1 to lamellipodia and cell-to-cell appositions [62] and in another study that demonstrated that S1P enhances the role of Cx in vasculoprotection [63]. Through these reported mechanisms, and possibly others, S1P maintains vascular functions such as regulation of transendothelial permeability [25, 62]. Surprisingly, the use of S1P to regenerate degraded HS did not improve the expression of Cx43 or the level of active communication. Instead, S1P turned out to be an important co-factor for exogenous HS. Regeneration of the GCX via the combination of S1P with exogenous HS resulted in both increased Cx43 at cell borders and greater movement of Lucifer Yellow dye across cells, indicating a recovery of structure and function of both GCX and gap junctions. In fact, HS/S1P-treatment over-recovered gap junctional dye spread, achieving a 1.4-fold increase compared to pre-degradation baseline conditions. Arguably, this gap junction hyperactivity (above baseline level) may signify some form of abnormality [64] or pathology. However, in a recent report by Jiang et al, it was demonstrated that high levels of Cx43-mediated gap junctional communication attenuate the degree of malignancy in multiple cancer cell lines [65]. Considering that cancer and vascular diseases share common pathology progression pathways [66], the results of the study conducted by Jiang et al can be extrapolated to suggest that over-recovery of interendothelial gap junction-mediated dye spread is not necessarily pathological and may actually be physiological beneficial. In future studies, a conscious effort must be made to identify and treat ECs with levels of S1P that ensure the stability of HS while at the same time confirming that restored interendothelial gap junction activity is within physiological limits. The mechanism of action of HS/S1P-treatment should also be clarified. For example, recovery of gap junction activity via HS/ S1P may involve non-Cx43 gap junction proteins (Cx40 and Cx37) as well as other junctional proteins (ZO-1, cadherins, and catenins, etc.) and other GAGs (chondroitin sulfate, sialic acid, and hyaluronic acid). This work is outside the scope of this study but of great relevance for fully understanding the relationship between GCX regeneration, Cx protein expression, and gap junction function.

**Fig 6:**
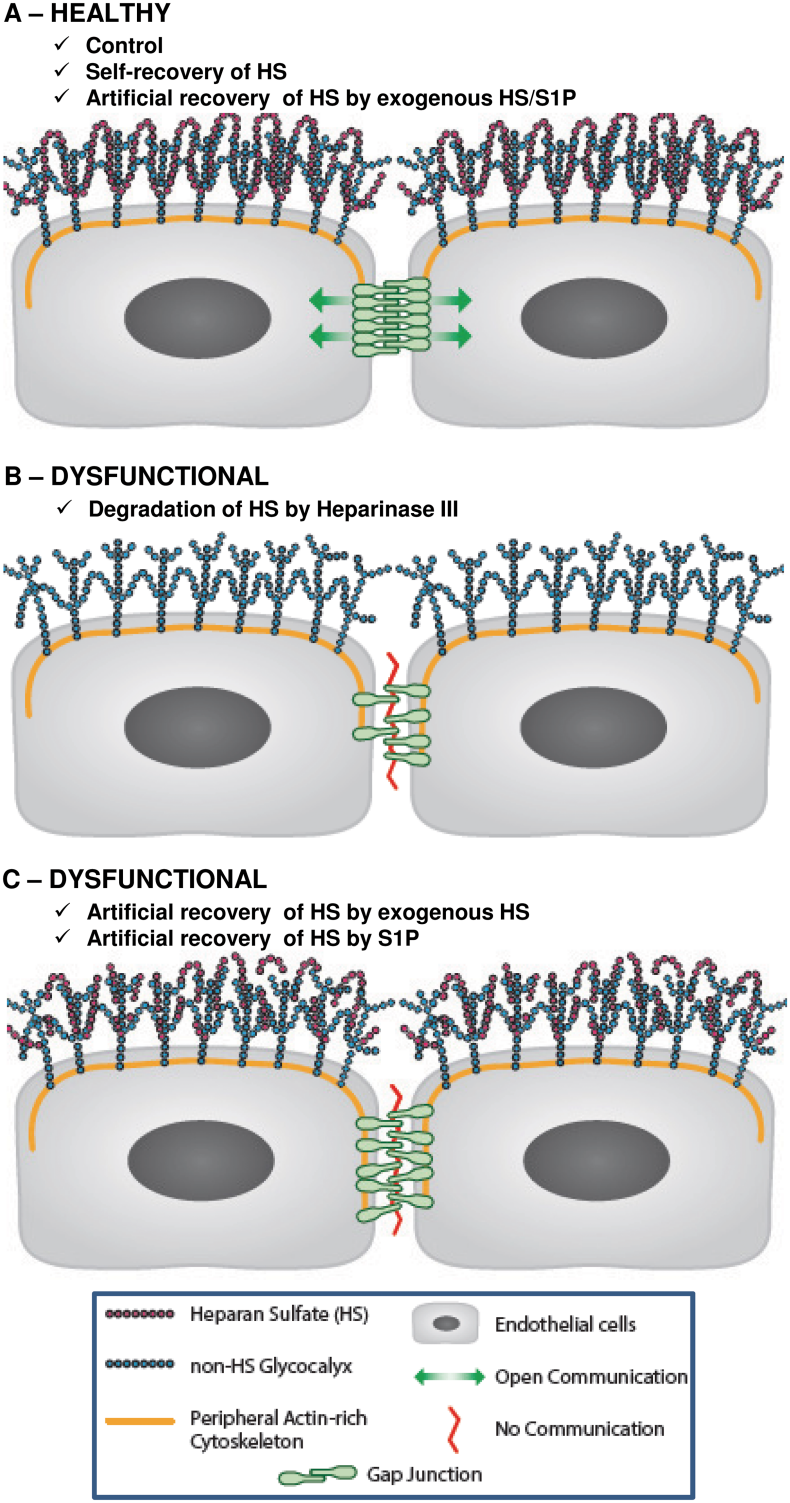
Conceptual hypothesis. **A**. Inter-endothelial gap junction is fully functional due to intact GCX (green arrows show Lucifer Yellow dye flux) in control conditions, when cells self-recover HS, or when HS is artificially recovered by treatment of cells with combination of exogenous HS and S1P. **B**. Degraded HS from GCX will cause the dislocation of Cx43 and malfunction of gap junction channels thereby preventing the movement of Lucifer Yellow dye across inter-endothelial gap junctions (red line indicates the improper functioning or blockage of gap junctions). **C**. Treatment of enzyme-treated cells with exogenous HS or S1P does not fully reset CX43 and/or recover intercellular gap junctional communication.

The findings of this work add fundamental knowledge to this understudied area and are encouraging for future therapeutics. As depicted in Fig 6, the key novel and compelling findings are: (i) removal of GCX-associated HS not only has the ability to alter organization of gap junction proteins, namely Cx43, but it also shuts down gap junction channel activity, and (ii) GCX repair by treating cells with exogenous HS and S1P restores gap junction protein placement which translates to the reactivation of gap junction channel activity.

To our knowledge, we are the first group to achieve functional GCX regeneration in ECs in a manner that effectively restores vasculoprotective EC communication within a short time frame. This current finding could translate to therapy for preventing atherosclerosis and motivates future development of new therapies targeted at the GCX to treat vascular disease. The ‘Holy Grail’ would be to discover innovative methods for regulating EC GCX enzymatic degradation and synthesis with subcellular Golgi and endoplasmic reticulum specificity and at vascular tree locations that are most relevant. In the meantime, in the absence of widely adopted approaches to reinforce the vascular GCX, implementation of our innovative approach in basic cell culture studies, pre-clinical in vivo studies, and, potentially, in clinical settings(Fig 5) will be highly significant to advance GCX knowledge and to better address endothelial-dependent disease processes.

## Supporting information

S1 Fig A. To quantify Cx43 coverage of RFPEC monolayer ImageJ automatically selected nine locations, as marked with red crosses, to randomize the cells that were quantified. B. To quantify gap junction mediated cell communication, cells were scratch-loaded (red-labeled cells only) with Lucifer Yellow dye, the dye spread to neighbors, and the neighboring cells that contained Lucifer yellow dye (green-labeled cells) were counted along lines perpendicular to the scratch.
(TIF)

S2 Fig A. Negative control for Cx43: Primary antibody specific to Cx43 was omitted in the immunostaining protocol to confirm the specificity of the antibody. No Cx43 was stained (blue stain indicates DAPI which stains the cell nucleus). B. Negative Control for HS: primary antibody targeting HS was omitted to confirm the specificity of the antibody. No HS was stained as observed both in the en face image and the orthogonal view (blue stain indicates DAPI staining for cell nucleus).
(TIF)

S3 Fig Dose-response test of the effect of Cytochalasin D on RFPEC actin filaments. A1. Control (untreated) EC sample in which blue is DAPI-stained cell nucleus and red is Alexa Fluor 647 conjugated phalloidin labeling the actin filaments. A2. Here, only the red channel is shown, to clarify that phalloidin-stained actin filaments are intact. B1. Treatment of EC sample with 50 nM of Cytochalasin D initiates the process of actin filament depolymerization, resulting in the some cytoskeletal instability. B2. The red channel is shown, to clarify phalloidin-stained actin filament deterioration. C1. Treatment of EC sample with 100 nM of Cytochalasin D totally arrests actin filament polymerization and results in rounded cell morphology. C2. In the red channel further deterioration of phalloidin-stained actin can be seen. (Scale bar is 20 μm and confocal microscopy magnification is 63X. Data was not quantitatively analyzed.)
(TIF)

S4 Fig A1. Phase contrast microscopy image of untreated RFPECs. A2. Lucifer yellow dye transfer to neighboring cells in untreated RFPEC samples. B1. Phase contrast image of enzyme (Hep III)-treated RFPEC. B2. Lucifer yellow dye transfer between cells, through gap junctions, was reduced in HepIII-treated cell populations. C1. Phase contrast microscopy image of RFPEC that were treated with exogenous HS and S1P after Hep lll to artificially regenerate the GCX. C2. Lucifer yellow dye transfer between neighboring cells was significantly recovered in comparison to Hep III-treated samples. D1. Phase contrast microscopy image of RFPEC after adding 50 nM of Cytochalasin D to disable F-actin in samples that were treated with exogenous HS and S1P after Hep III to artificially regenerate the GCX. D2. Adding 50 nM of Cytochalasin D for the last 30 minutes of the GCX regeneration period reduced Lucifer yellow dye transfer that resulted from treatment with exogenous HS and S1P. E1. Phase contrast microscopy image of RFPEC after adding 100 nM of Cytochalasin D to disable F-actin in samples that were treated with exogenous HS and S1P after Hep III to artificially regenerate the GCX. E2. Adding 100 nM of Cytochalasin D for the last 30 minutes of the GCX regeneration period caused the highest reduction in Lucifer yellow dye transfer that resulted from treatment with exogenous HS and S1P. F1. Phase contrast microscopy image of RFPEC exposed for 30 minutes to dimethyl sulfoxide (DMSO), the Cytochalasin D delivery vehicle, after treatment with exogenous HS and S1P to artificially regenerate GCX following pre-treatment with GCX-degrading HepIII. F2. DMSO alone has some effect on cell-to-cell communication, which clarifies the relative effects Cytochalasin D induced actin cytoskeleton arrest. Lucifer yellow dye transfer between neighboring cells is clearly impacted by 50 nM Cytochalasin D when comparing the results shown in F2 versus E2 and more impacted by 100 nM Cytochalasin D when the results in F2 versus D2 are compared. (Note: Scale bar is 100 μm, and microscope magnification is 10X. A portion of this data was quantitatively analyzed, as reported in main article. A portion of this data was analyzed qualitatively, because it was collected outside of the scope of the main project and only as part of a pilot experiment to support the conclusions of the main study.)
(TIF)

## Abbreviations

Cx: Connexin
DMEM: Dulbecco’s Modified Eagle Medium
ECs: Endothelial Cells
FBS: Fetal Bovine Serum
GAGs: Glycosaminoglycans
GCX: Endothelial Glycocalyx
Hep III: Heparinase III
HS: Heparan Sulfate
NDST: N-deacetylase/N-sulfotransferase
PS: Penicillin-Streptomycin
RFPEC: Rat Fat Pad Endothelial Cells
S1P: Sphingosine 1-Phosphate
ZO-1: Zona Occludin 1
