# Supplementary figures and images for: Regeneration of glycocalyx by heparan sulfate and sphingosine 1-phosphate restores inter-endothelial communication
Source: PLoS One. 2017 Oct 12;12(10):e0186116. doi: 10.1371/journal.pone.0186116 (PMC5638341; doi:10.1371/journal.pone.0186116)

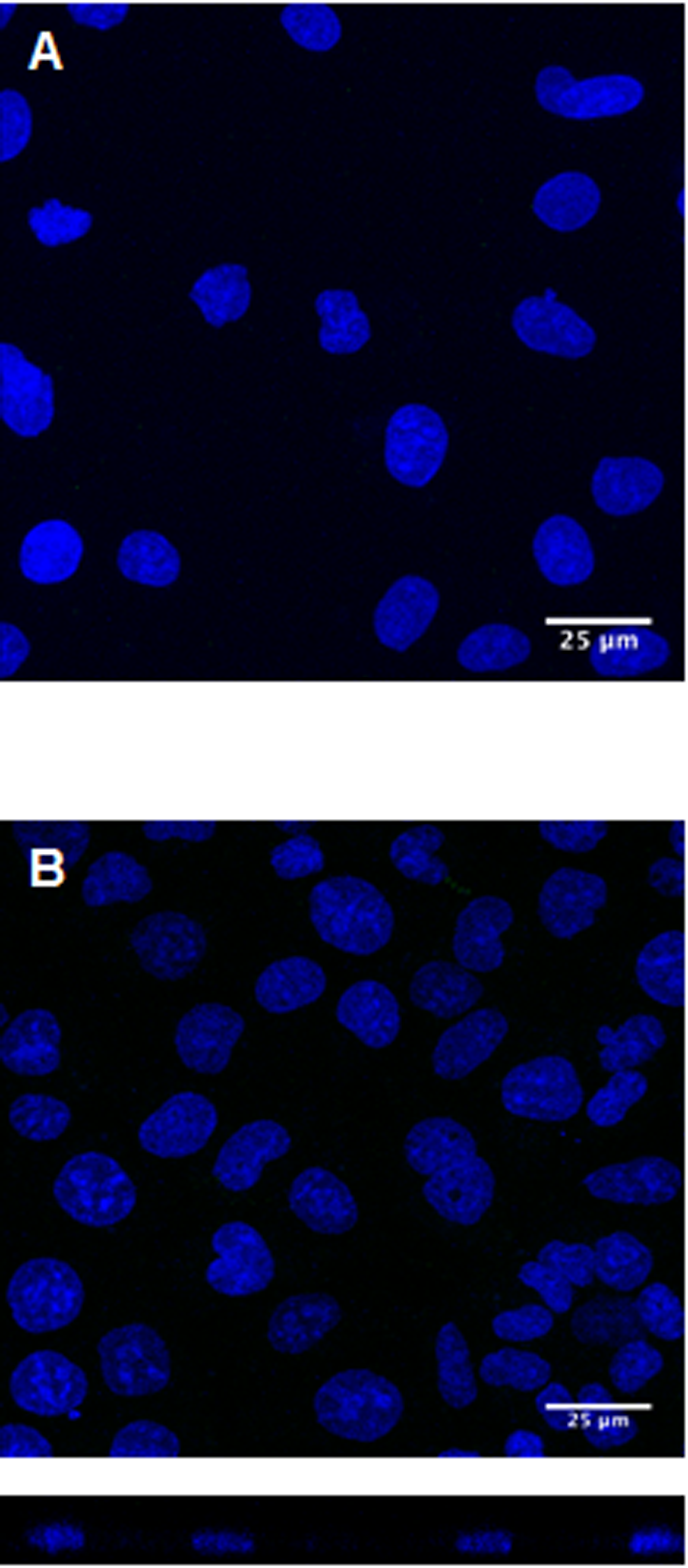

Supplement: S1 Fig — A. To quantify Cx43 coverage of RFPEC monolayer ImageJ automatically selected nine locations, as marked with red crosses, to randomize the cells that were quantified. B. To quantify gap junction mediated cell communication, cells were scratch-loaded (red-labeled cells only) with Lucifer Yellow dye, the dye spread to neighbors, and the neighboring cells that contained Lucifer yellow dye (green-labeled cells) were counted along lines perpendicular to the scratch. (TIF) [file pone.0186116.s001.tif]

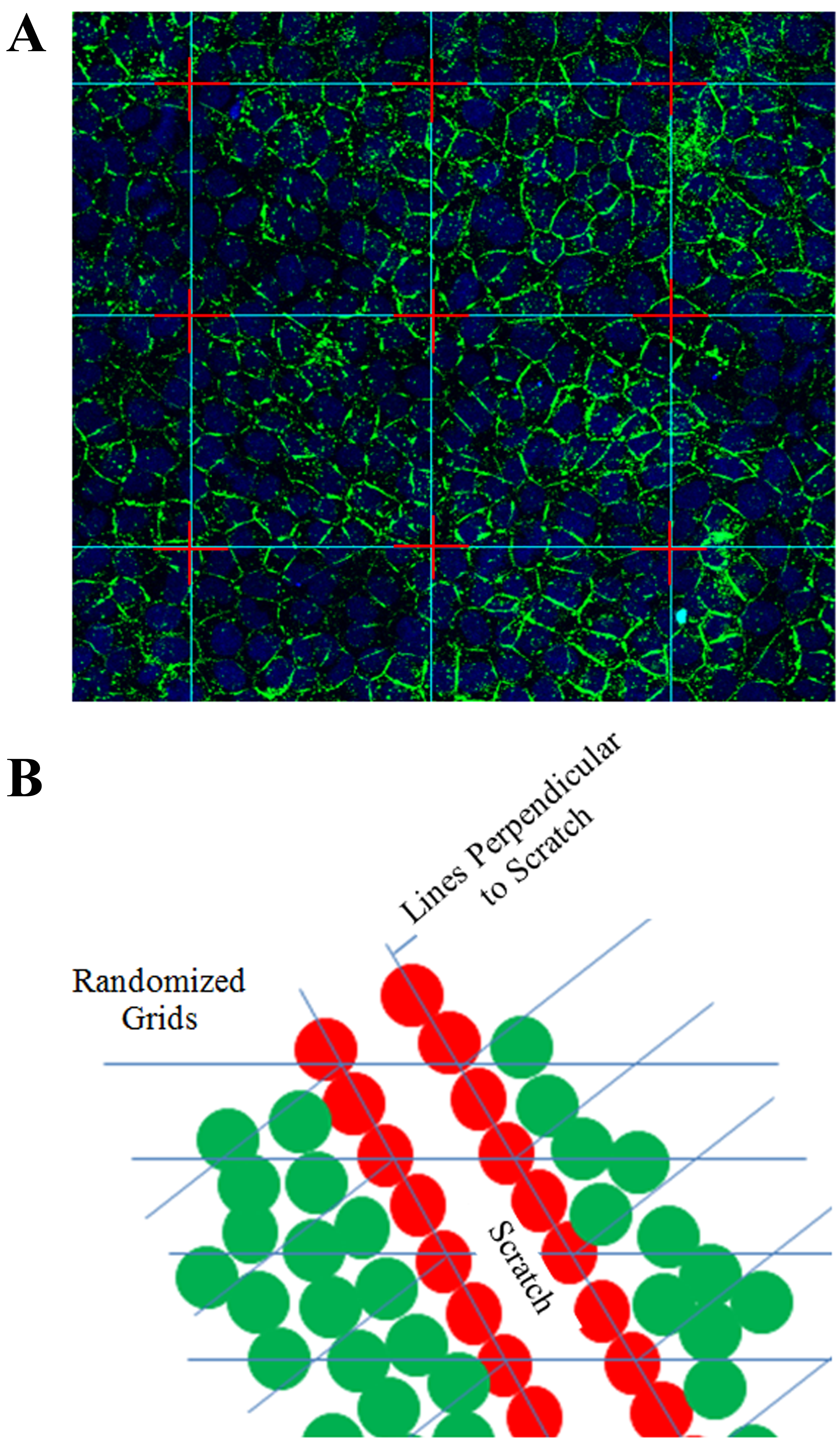

Supplement: S2 Fig — A. Negative control for Cx43: Primary antibody specific to Cx43 was omitted in the immunostaining protocol to confirm the specificity of the antibody. No Cx43 was stained (blue stain indicates DAPI which stains the cell nucleus). B. Negative Control for HS: primary antibody targeting HS was omitted to confirm the specificity of the antibody. No HS was stained as observed both in the en face image and the orthogonal view (blue stain indicates DAPI staining for cell nucleus). (TIF) [file pone.0186116.s002.tif]

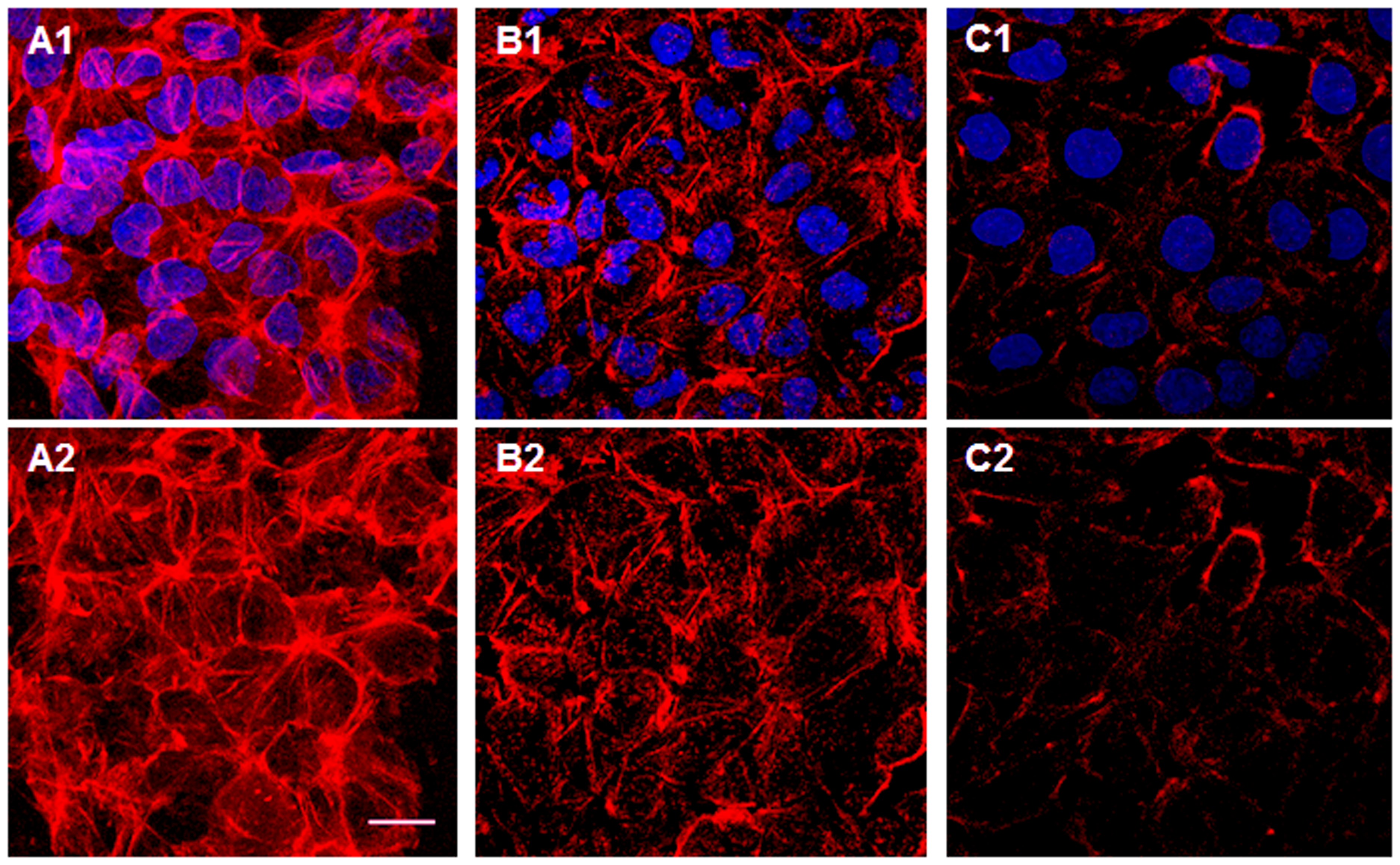

Supplement: S3 Fig — A1. Control (untreated) EC sample in which blue is DAPI-stained cell nucleus and red is Alexa Fluor 647 conjugated phalloidin labeling the actin filaments. A2. Here, only the red channel is shown, to clarify that phalloidin-stained actin filaments are intact. B1. Treatment of EC sample with 50 nM of Cytochalasin D initiates the process of actin filament depolymerization, resulting in the some cytoskeletal instability. B2. The red channel is shown, to clarify phalloidin-stained actin filament deterioration. C1. Treatment of EC sample with 100 nM of Cytochalasin D totally arrests actin filament polymerization and results in rounded cell morphology. C2. In the red channel further deterioration of phalloidin-stained actin can be seen. (Scale bar is 20 μm and confocal microscopy magnification is 63X. Data was not quantitatively analyzed.) (TIF) [file pone.0186116.s003.tif]

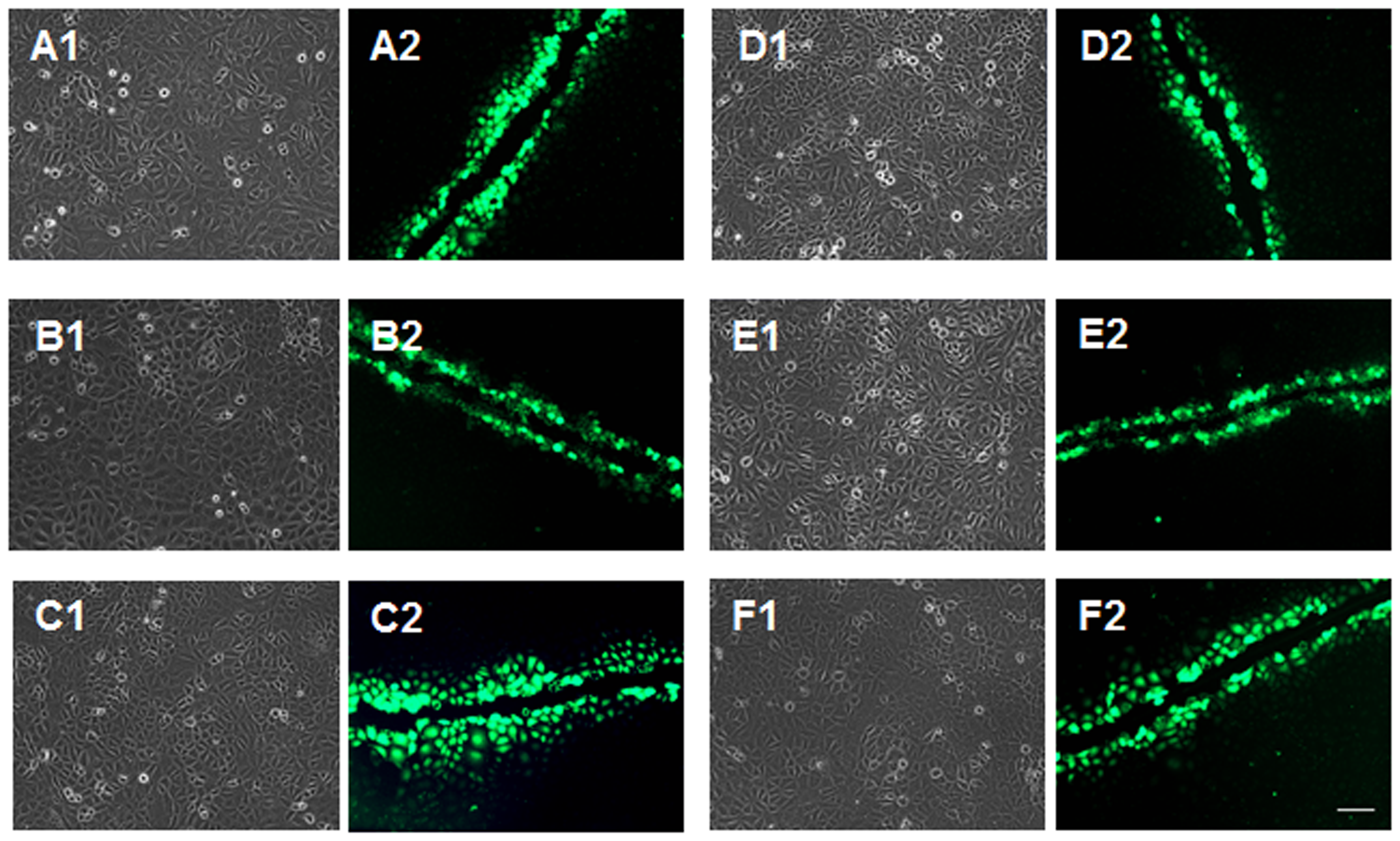

Supplement: S4 Fig — A1. Phase contrast microscopy image of untreated RFPECs. A2. Lucifer yellow dye transfer to neighboring cells in untreated RFPEC samples. B1. Phase contrast image of enzyme (Hep III)-treated RFPEC. B2. Lucifer yellow dye transfer between cells, through gap junctions, was reduced in HepIII-treated cell populations. C1. Phase contrast microscopy image of RFPEC that were treated with exogenous HS and S1P after Hep lll to artificially regenerate the GCX. C2. Lucifer yellow dye transfer between neighboring cells was significantly recovered in comparison to Hep III-treated samples. D1. Phase contrast microscopy image of RFPEC after adding 50 nM of Cytochalasin D to disable F-actin in samples that were treated with exogenous HS and S1P after Hep III to artificially regenerate the GCX. D2. Adding 50 nM of Cytochalasin D for the last 30 minutes of the GCX regeneration period reduced Lucifer yellow dye transfer that resulted from treatment with exogenous HS and S1P. E1. Phase contrast microscopy image of RFPEC after adding 100 nM of Cytochalasin D to disable F-actin in samples that were treated with exogenous HS and S1P after Hep III to artificially regenerate the GCX. E2. Adding 100 nM of Cytochalasin D for the last 30 minutes of the GCX regeneration period caused the highest reduction in Lucifer yellow dye transfer that resulted from treatment with exogenous HS and S1P. F1. Phase contrast microscopy image of RFPEC exposed for 30 minutes to dimethyl sulfoxide (DMSO), the Cytochalasin D delivery vehicle, after treatment with exogenous HS and S1P to artificially regenerate GCX following pre-treatment with GCX-degrading HepIII. F2. DMSO alone has some effect on cell-to-cell communication, which clarifies the relative effects Cytochalasin D induced actin cytoskeleton arrest. Lucifer yellow dye transfer between neighboring cells is clearly impacted by 50 nM Cytochalasin D when comparing the results shown in F2 versus E2 and more impacted by 100 nM Cytochalasin D when the results [file pone.0186116.s004.tif]
